# A case of Vp4 hepatocellular carcinoma with tumor thrombosis extending into the confluence of the splenic/portal vein achieved a good prognosis with emergent hepatectomy and postoperative adjuvant therapy with lenvatinib

**DOI:** 10.1186/s12957-022-02740-w

**Published:** 2022-09-03

**Authors:** Hiroyuki Kato, Yukio Asano, Masahiro Ito, Satoshi Arakawa, Masahiro Shimura, Daisuke Koike, Takayuki Ochi, Hironobu Yasuoka, Toki Kawai, Takahiko Higashiguchi, Hiroki Tani, Yoshiki Kunimura, Yuka Kondo, Hidetoshi Nagata, Harunobu Sato, Akihiko Horiguchi

**Affiliations:** Department of Gastroenterological Surgery, Fujita Health University School of Medicine, Bantane Hospital, 3-6-10 Otobashi Nakagawa Ward Nagoya, Nagoya, Aichi 454-8509 Japan

**Keywords:** Vp4, Tumor thrombosis, Lenvatinib

## Abstract

In this report, we describe a case of highly advanced hepatocellular carcinoma with tumor thrombosis extending into the main portal vein of the pancreas that was successfully treated with adjuvant lenvatinib after right hepatic resection with thrombectomy. A 70-year-old woman was referred from the clinic because of elevated hepatobiliary enzymes. The patient was positive for the hepatitis B virus antigen at our hospital. The tumor markers were highly elevated with alpha-fetoprotein (14.5 U/mL) and protein induced by vitamin K absence (PIVKAII) (1545 ng/mL), suggesting hepatocellular carcinoma. Dynamic abdominal computed tomography showed an early enhanced tumor approximately 6 cm in size and portal vein tumor thrombosis filling the main portal vein, but not extending into the splenic or superior mesenteric vein (SMV). On magnetic resonance imaging 1 week after CT, portal vein tumor thrombosis had extended to the confluence of the splenic vein with the SMV, indicating rapid tumor growth. Thus, we performed emergent right hepatectomy with tumor thrombectomy. Postoperatively, we treated the patient with lenvatinib for a tumor reduction surgery. Fortunately, the patient was alive 2 years postoperatively without recurrence. This case report suggests that a favorable outcome may be achieved with multidisciplinary treatment including resection and postoperative treatment with lenvatinib.

## Introduction

The prognosis of hepatocellular carcinoma (HCC) with portal vein tumor embolization is extremely poor, especially for Vp4 HCC with thrombosis in the main trunk of the portal vein. The indications for surgery in such cases are yet to be established and are dependent on the institution. Herein, we describe a case of highly advanced HCC with tumor thrombosis extending into the main portal vein of the pancreas that was successfully treated with adjuvant lenvatinib after right hepatic resection with thrombectomy.

## Case report

**Fig. 1:**
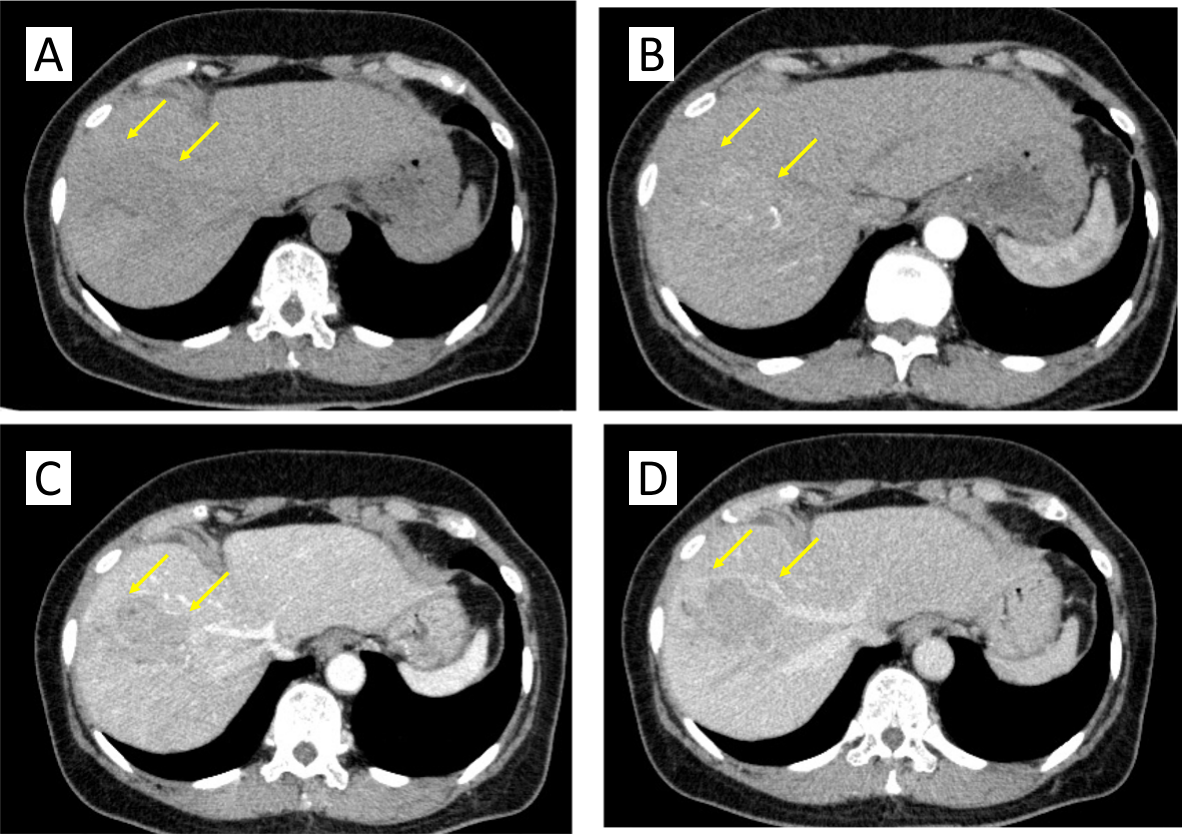
Preoperative dynamic enhanced computed tomography. **A** Plain image. **B** Arterial phase. **C** Portal phase. **D** Equivalent phase. A 6-cm early enhanced, unclear boundary tumor was seen in the anterior section of the liver, segment 8, which infiltrated the middle hepatic vein

**Fig. 2:**
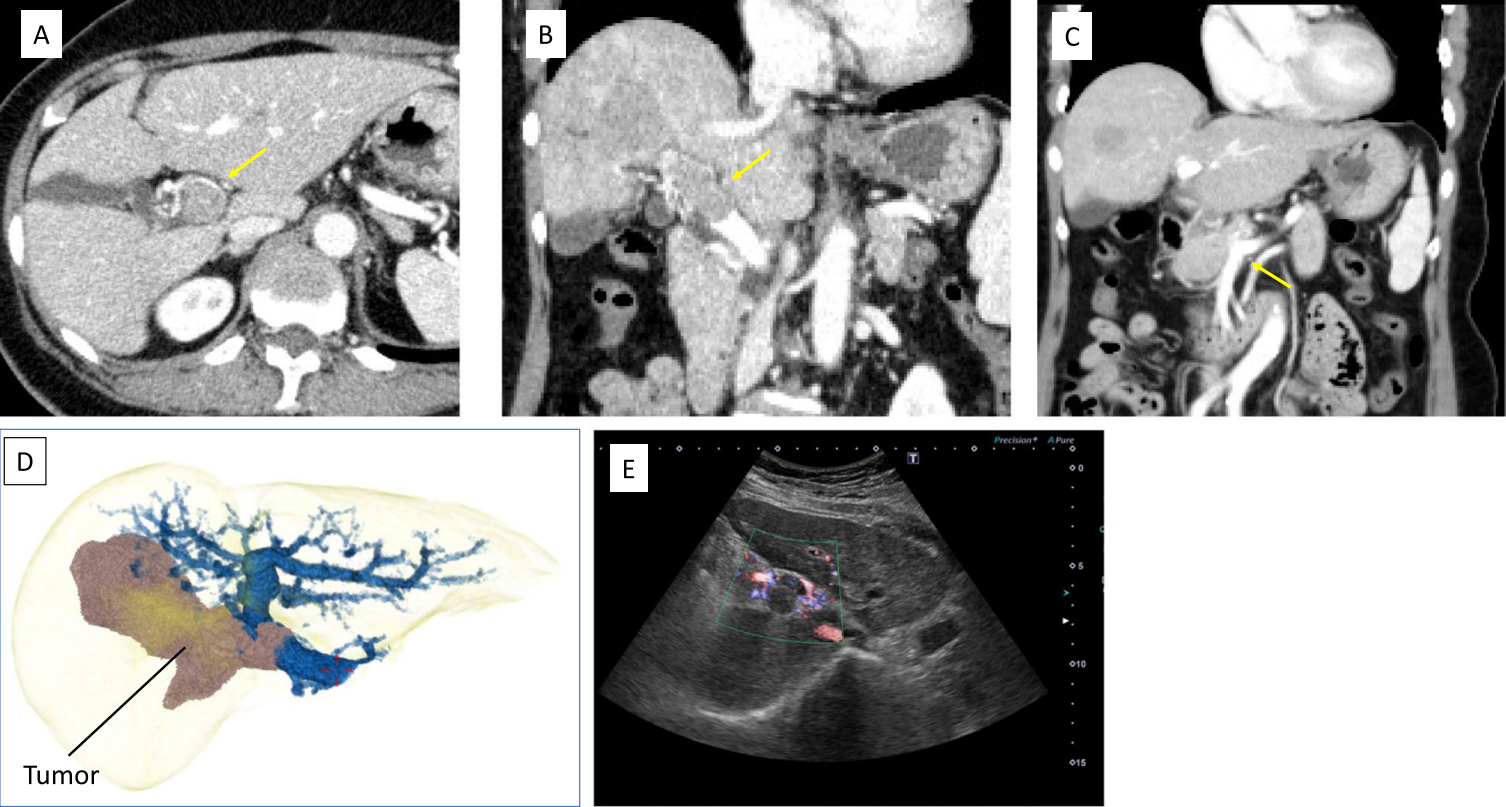
Preoperative imaging evaluating the tumor thrombus of the portal vein at the time of admission. **A** Axial image of the enhanced computed tomography (CT) image. **B** Coronal image. **C** Coronal image of the enhanced CT showing that the confluence of the superior mesenteric vein and splenic vein is intact. **D** Three-dimensional imaging showing the association between the tumor and portal vein. **E** Abdominal ultrasonography showing that barely any portal blood flow is present

**Fig. 3:**
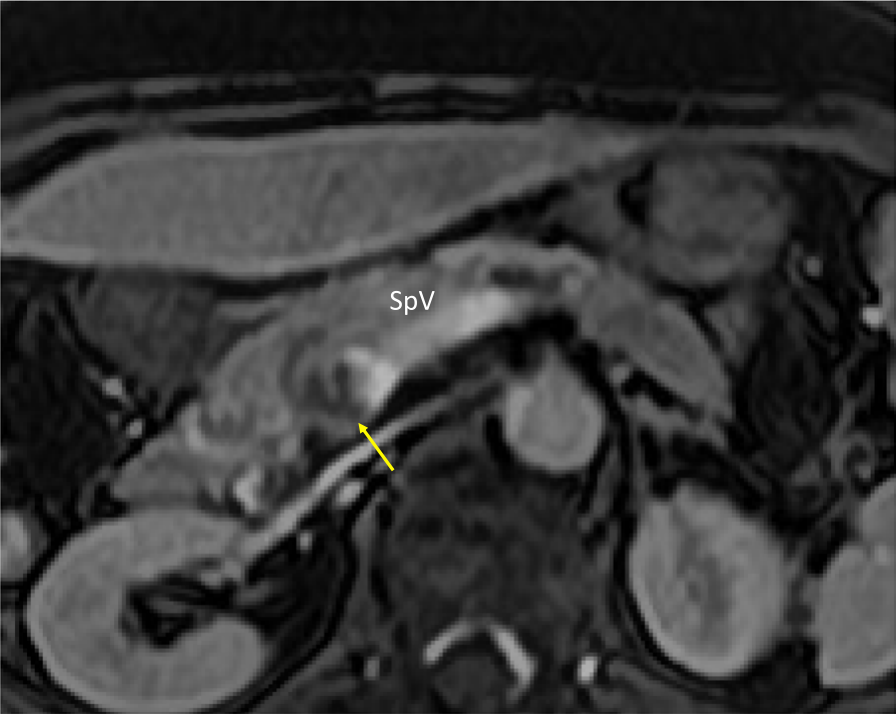
Magnetic resonance imaging 1 week after contrast-enhanced computed tomography showing the tumor emboli extending to the confluence of the superior mesenteric vein/splenic vein

**Fig. 4:**
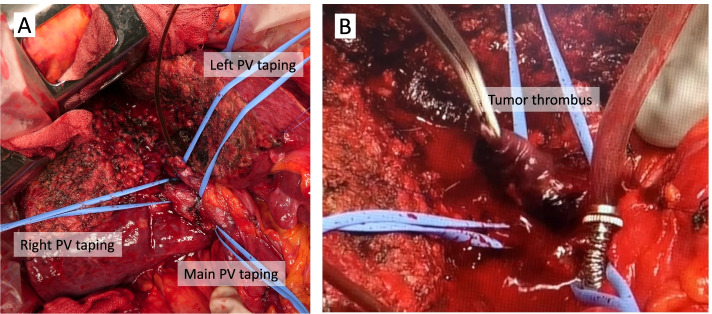
Intraoperative findings. **A** Picture in which the right, left, and main portal veins are encircled after liver resection. **B** The picture in which tumor thrombectomy is performed

**Fig. 5:**
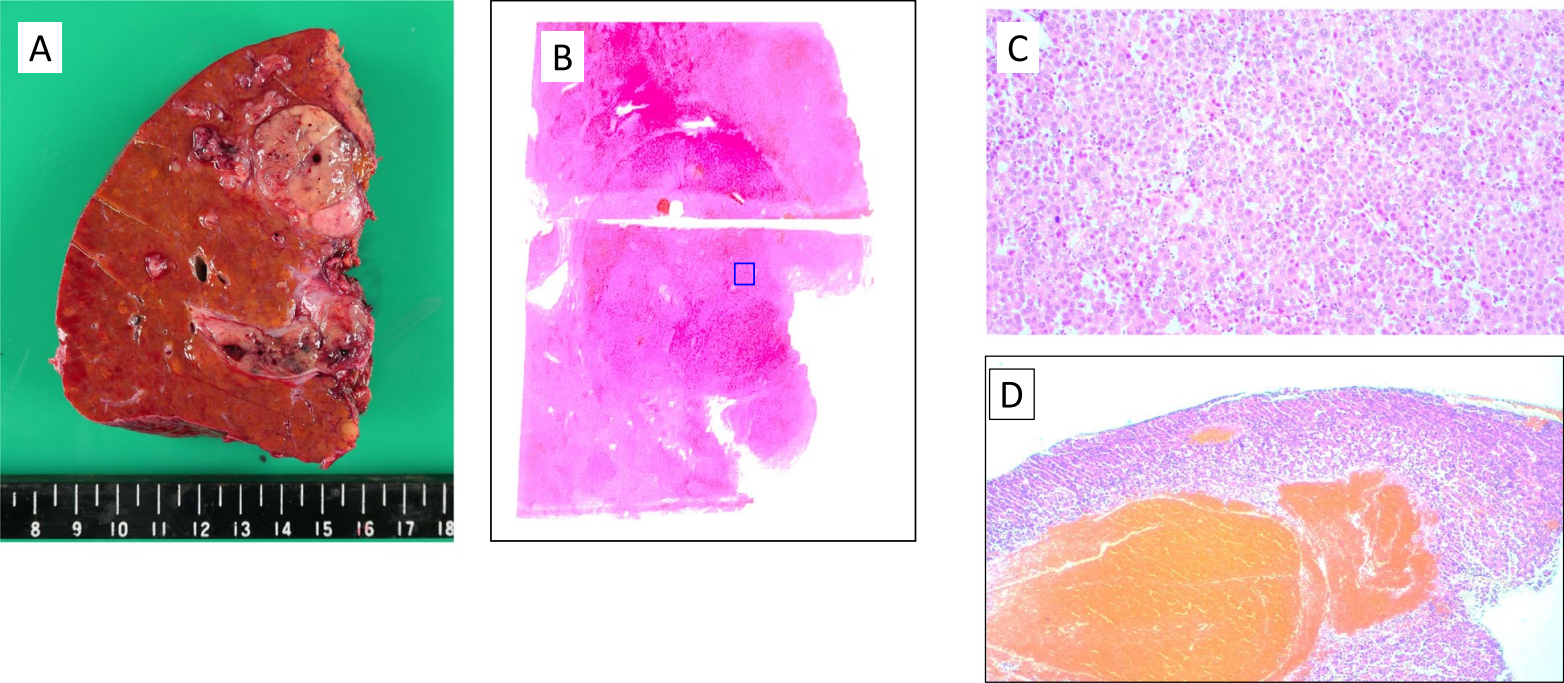
Macro- and microscopic findings of the resected specimen. **A** Macroscopic finding. **B** Loupe finding. **C** Microscopic findings of the tumor. **D** Microscopic findings of the tumor thrombus. The final diagnosis is 35 × 25 mm hepatocellular carcinoma, moderately differentiated type. Vp4, vv0, va0, b0, p0, and surgical margin-negative

A 70-year-old woman was referred from the clinic because of elevated hepatobiliary enzymes. She was found positive for hepatitis B virus antigen at our hospital and underwent close examination at the Department of Gastroenterology. After a thorough examination, she was found to have elevated levels of alpha-fetoprotein (AFP) and protein induced by vitamin K absence (PIVKA-II) and was referred to our department for further evaluation. According to the blood test at the time of admission, hepatobiliary enzymes were mildly elevated, and tumor markers were highly elevated, with AFP of 14.5 U/mL and PIVKAII of 1545 ng/mL, suggesting HCC. Dynamic abdominal computed tomography (CT) showed an early enhanced tumor, approximately 6 cm in size, infiltrating the middle hepatic vein in segment 8 of the liver (Fig. 1). Tumor thrombosis was observed in the main portal vein (Fig. 2A). The coronal section of the CT scan showed portal vein tumor thrombosis filling the main portal vein but not extending into the splenic vein or superior mesenteric vein (SMV) (Fig. 2B, C). Doppler ultrasonography showed that the flow in the portal vein was barely preserved (Fig. 2D). Magnetic resonance imaging (MRI) 1 week after the CT showed that the portal vein tumor thrombosis had extended to the confluence of the splenic vein with the SMV, indicating rapid tumor growth (Fig. 3). In terms of preoperative liver function, the ICG clearance rate (ICGK) and ICGR15 were 0.164 and 8%, respectively. Meanwhile, the estimated remnant liver volume was 64%, with a remnant ICGK value of 0.103, indicating that the patient could tolerate surgery. Thus, we performed emergent right hepatectomy with tumor thrombectomy because the rapid extension of the tumor thrombosis was considered life-threatening. Figure 4 shows the surgical findings. First, the inferior border of the pancreas was dissected, and the SMV was taped, followed by taping of the splenic vein. Liver resection was performed after taping of the right hepatic hilar plate. The right hepatic duct and artery were divided, and the right, left, and main portal veins were taped. While clamping the left portal vein, SMV, and splenic vein, the right portal vein was incised by 1 cm, and the tumor thrombosis was removed as carefully as possible. After thrombectomy, intraoperative ultrasonography showed no residual tumor thrombus, the right portal vein was divided, and the stump was closed with 6–0 Prolene. Blood loss was 3325 g, and the operation time was 384 min. The resected specimen revealed that the tumor was a 35 × 25 mm HCC, moderately differentiated type, Vp4, vv0, va0, b0, p0, and surgical margin-negative (Fig. 5).

**Fig. 6:**
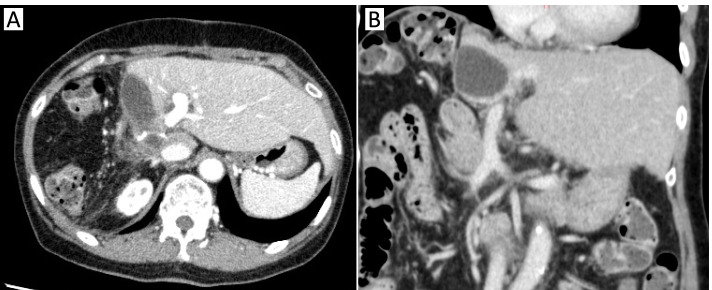
Latest enhanced CT scan done one and half years after the surgery, showing that there is no residual tumor and recurrence

Postoperatively, the patient had no complications and was discharged on postoperative day 20. After a joint conference between gastroenterology and surgery, we decided to treat the patient with lenvatinib postoperatively because we considered this surgery to be a tumor-reduction surgery rather than a radical surgery. Lenvatinib was initiated at 8 mg/day and continued for 6 months. Subsequently, the dose was reduced to 4 mg/day and is continuing now. The patient visited the outpatient clinic every 2 months. Tumor markers such as AFP and PIVKAII were measured at that time. Enhanced CT was performed every 4 months to check for recurrence. Fortunately, the patient was alive 2 years postoperatively without recurrence (Fig. 6).

## Discussion

Many studies [1–4] have shown that portal vein thrombosis is the strongest negative prognostic factor for HCC. Even when tumor thrombosis is localized within the primary branch of the portal vein (up to Vp3), their 5-year survival rates are around 8–35% [5]. However, if the tumor thrombosis extends to the main trunk of the portal vein (Vp4), the prognosis is extremely poor, and surgical resection is generally not indicated; even if resection is intended, more than 60% of cases are insufficiently resected, and the median survival time after resection is reported to be less than 1 year with a 90-day mortality rate of 8.2% [5]. Therefore, it is difficult to improve the prognosis of such highly advanced HCC by surgical resection alone, and multimodality treatment using new molecular targeted drugs may be an option to improve prognosis.

The usefulness of lenvatinib for unresectable HCC with portal vein tumor thrombosis has been reported in recent years [6–8]; Chuma et al. [9] reported favorable results in patients with Child–Pugh (CP) grade A unresectable HCC. In a recent study, they reported that the response rate to lenvatinib in patients with CP grade A unresectable HCC was significantly higher (26.7%) than in those with grade B unresectable HCC (0%). In Vp4 HCC, the median progression-free survival was worse in patients with CP grade B (57 days) than in those with CP grade A (137 days, *p* = 0.0462). Regarding multimodal treatment using a combination of surgery and lenvatinib, there have been several reports of patients with Vp4 HCC with pulmonary metastases or tumor thrombosis extending to the contralateral side, in whom lenvatinib was administered, and the tumor shrank, resulting in conversion hepatectomy to the primary tumor [7, 10]. In the present case, the patient was asymptomatic, and her performance status was well preserved. As shown by ultrasonography (Fig. 3), the main trunk of the portal vein was almost occluded, but intrahepatic portal vein flow was barely maintained. Even though there was no metastatic region, portal vein tumor thrombosis progressed rapidly within a week; therefore, emergency hepatic resection and thrombectomy were performed the day after the MRI. The patient provided informed consent for surgery, and the possibility of intraoperative death or serious complications was fully explained to the patient. The thrombosis was removed directly from the main trunk of the portal vein by aspiration with a small suction tube.

Fortunately, no postoperative complications occurred, and the postoperative treatment was promptly initiated. To the best of our knowledge, this is the first case of Vp4 HCC treated with lenvatinib after hepatectomy with tumor thrombectomy, which resulted in a favorable prognosis.

## Conclusions

Although the prognosis of Vp4 HCC is extremely poor in previous reports, this case suggests that a favorable outcome may be achieved with multidisciplinary treatment, including resection and postoperative treatment.

## Data Availability

All the data generated or analyzed during this study are included within the article.
